# Straatsma Syndrome and cataract: case report and review of the literature

**DOI:** 10.22336/rjo.2023.67

**Published:** 2023

**Authors:** Casado-Pelaez Blanca, Pascual-Camps Isabel, Inat-Moreno Sergio, Congost-Laguna Candela, Barranco-Gonzalez Honorio, España-Gregori Enrique

**Affiliations:** * Department of Ophthalmology, La Fe University and Polytechnic Hospital, Valencia, Spain

**Keywords:** Straatsma Syndrome, myopia, myelinated retinal nerve fibers, amblyopia, cataract

## Abstract

Straatsma Syndrome is known as unilateral myopia, amblyopia, and myelinated retinal nerve fibers (MRNF). The syndrome can be associated with other findings such as nystagmus, strabismus, and optic nerve hypoplasia among others. However, no cases associated with cataract have been reported. The visual prognosis depends on the myelinated retinal nerve fibers extension, the early amblyopia therapy, and the coexistence of other signs. We present the case of a 4-year-old girl with Straatsma Syndrome and cataract in the left eye. Despite the cataract surgical treatment with the refractive error correction and the amblyopia therapy, no visual improvement has been reported.

**Abbreviations:** MRNF = Myelinated retinal nerve fibers, LE = Left eye, PD = Prism dioptres, BCVA = Best-corrected visual acuity, RE = Right eye, HM = Hand movement, CF = Counting fingers

## Introduction

The triad composed of unilateral myelinated retinal nerve fibers (MRNF), axial myopia, and amblyopia is known by the name of Straatsma Syndrome. Although it is true that in 1979 the syndrome was discovered by Straatsma et al. [**1**] as MRNF, myopia, amblyopia, and strabismus, nowadays the scientific literature does not include strabismus as one of the necessary criteria [**2**]. Generally, it is unilateral, but bilateral cases have been described [**3**] and are associated with hyperopia, which is known as reverse Straatsma Syndrome. Other described associated signs are heterochromia iridium [**4**], nystagmus [**2**], and optic nerve hypoplasia. To our knowledge, no cases associated with cataract have been reported. The degree of affectation of visual acuity is related to the extension of MRNF, the coexistence of strabismus or nystagmus, and macular involvement, among others. The correction of anisometropia and the early treatment of amblyopia are the central treatments; however, the results are usually not satisfactory [**5**,**6**].

This article presents the case of a patient with classic unilateral Straatsma Syndrome which is also associated with cataract in the affected eye. 

## Case report

A 4-year-old girl was referred to our clinic with a previous emergency room report in which they described a sudden deviation of the left eye (LE) two weeks before (alternating 40 prism dioptres (PD) esotropia with episodes of orthotropia), along with cataract of the LE. 

**Fig. 1:**
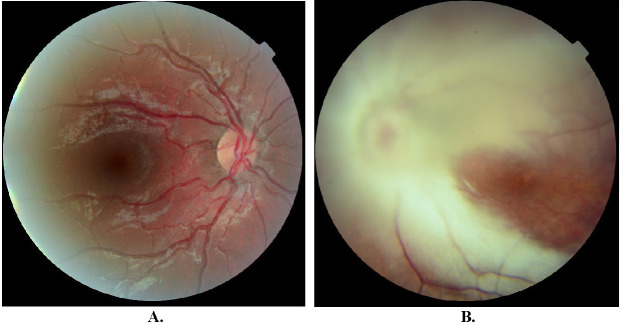
**A.** Normal fundus of the right eye; **B.** Left fundus showing extensive myelinated retinal nerve fiber in both temporal vascular arcades with minimum macular sparing and hypoplastic optic nerve

On examination, the patient’s best-corrected visual acuity (BCVA) was +0,3 logMAR in the right eye (RE), and hand movement (HM) at 20 centimeters in LE; normal anterior segment in RE and anterior polar cataract along with posterior subcapsular cataract (more accentuated in the superonasal quadrant) in LE. Cycloplegic refraction was +1.50 dioptre sphere with a -0.25-dioptre cylinder at 16° in the RE and -17 dioptre sphere with a -1.50-dioptre cylinder at 13° in LE. A dilated fundus examination was performed, showing a normal eye fundus in RE (**Fig. 1 A**). In LE, extensive myelinated retinal nerve fibers were visualized in both temporal vascular arcades with minimum macular sparing, in addition to the hypoplastic optic nerve and attached retina (**Fig. 1 B**). No other systemic findings were detected.

Surgery was scheduled to extract the cataract. A lens was inserted to correct the myopia which left the patient with a degree of physiological hypermetropia adjusted to her age. After surgery, the patient was examined, showing good surgical results with a free pupillary axis and clear cornea. The final refraction obtained after surgery was +2,50 dioptre sphere with -1.00 dioptre cylinder at 155° in LE; at that time, the BCVA in the RE was +0,3 logMAR and counting fingers (CF) in the LE. Occlusion therapy was prescribed for anisometropic amblyopia in the LE. The patient followed a 1 hour/day occlusion for three months. The patching regimen was then increased to 4 hours/day for four months until the present. The last consultation revealed a BCVA of +1 logMAR in the left eye and +0,2 logMAR in the right eye. Left eye esotropia episodes disappeared achieving orthotropia. Fundus alterations have remained stable.

## Discussion

To our knowledge, no case has been published until present in the scientific literature, in which the Straatsma Syndrome and ipsilateral cataract coexist, this being the first case reported. It is estimated that between 7.40 and 15.50% of cases of infant blindness worldwide are due to congenital cataracts [**7**], with no significant differences regarding gender or unilaterality [**8**]. However, the prevalence of cataracts in infancy is relatively low: every 10,000 children between 0.32 and 22.90 suffer this malformation [**7**]. 

The diagnosis, surgical treatment, and intensive and early visual therapy are fundamental in the visual prognosis. Despite this, the results are not always favorable [**9**], with better results being obtained in bilateral cataracts than in unilateral cases [**10**]. In a study of 57 eyes affected by bilateral and unilateral cataracts, it was observed that even though 80.71% of the cases of congenital cataract improved their VA after surgery, only 21% of them obtained a postsurgical VA greater than or equal to +0,3 logMAR [**11**]. In another study of 20 cases of unilateral congenital cataract, only 6 of them obtained a VA between +1 - +0.4 logMAR after surgery, and the other 16 maintained a VA of CF or less [**12**]. 

The myelinated retinal nerve fiber is present in approximately 1% [**4**] of the population. Although the literature establishes a prevalence between 0.03 and 10%, there are insufficient references to establish an exact margin [**7**]. In most cases, MRNF is an isolated and asymptomatic finding, but it may appear in the context of a syndrome, such as the case of Straatsma Syndrome and the Gorlin-Goltz syndrome [**13**]; or associated with other ocular disorders [**14**], such as strabismus, retinal membrane, persistent foetal vasculature, and even cephalic disorders [**4**], all of them worsening the visual prognosis. There are numerous associations in the scientific literature with the Straatsma Syndrome, such as nystagmus, heterochromia iridum, strabismus, retinal vascular, retinal membrane, optic nerve hypoplasia, persistent foetal vasculature, cephalic disorders [**2**,**4**]; however, until present no cataract association has been described, being the only case reported. 

The amblyopia generated in the Straatsma Syndrome is due to the degree of the anisometropia and the extent of the MRNF and its macular involvement, being an asymptomatic finding in so many cases [**4**]. According to some studies, patients with 5 hours or less of retinal involvement showed the best improvement, and patients with 9 hours or more of retinal involvement showed the worst result [**5**]. It has been postulated that an organic etiology may also contribute to the amblyopia and the poor response to occlusion therapy that Straatsma Syndrome patients obtain, having cases of Straatsma Syndrome in which a loss or disturbance of the ellipsoid zone have been reported [**14**]. 

Despite the early treatment of amblyopia, the sensorial deprivation, secondary to the cataract in an eye already affected by high myopia and MRNF, drastically darkens the visual prognosis. In our case, the patient’s BCVA improved from HM at 20 centimeters to +1 logMAR in the LE at eight months after the initial presentation.

## Conclusion

In conclusion, early amblyopia therapy is fundamental in cases of both Straatsma Syndrome and infantile cataracts to achieve BCVA. Furthermore, this case revealed an unusual association between congenital cataract and Straatsma Syndrome that has not been reported before, which may be caused by a common etiopathogenic mechanism. 

**Conflict of Interest Statement**

The authors declare that they have no conflict of interest.

**Informed Consent and Human and Animal Rights Statement**

Informed consent was obtained from the parents of the individual included in this study.

**Authorization for the use of human subjects**

Ethical approval: The research related to human use complied with all relevant national regulations and institutional policies, followed the tenets of the Helsinki Declaration, and was approved by the Ethics Committee of La Fe University and Polytechnic Hospital of Valencia, Valencia, Spain.

**Acknowledgments**

None.

**Sources of Funding**

None.

**Disclosures**

None.
